# Move Right along in This Line

**Published:** 1897-07

**Authors:** 


### MOVE RIGHT ALONG IN THIS LINE.

WE are glad to announce that about July 15th Professor John W. Adams, of the Veterinary Department of the University of Pennsylvania, will bring forth the projected work on the *First Principles of Shoeing*. It will cover in a measure the place of instructor in horse-shoeing, so far as this may be accomplished by a work of this kind. The book will contain some one hundred and forty pages of reading-matter, and one hundred and forty-four illustrations. It will be placed on the market at a popular price.

WHEN the different States shall take up in their Legislatures the need of better sanitary measures in railroad live-stock transportation, compelling railroads within their borders to regularly and thoroughly disinfect their cars, there will be fewer losses to shippers and breeders, whose cup of grievances has been full to overflowing the past two years.

---

SOME 27,000 horses were inspected in Montana during the past two months, most of those shipped from the State being sent to Dakota. Every State has an equal amount of veterinary service, if the proper organization of its sanitary service was inaugurated.

